# Root architecture governs plasticity in response to drought

**DOI:** 10.1007/s11104-018-3824-1

**Published:** 2018-10-25

**Authors:** Ellen L. Fry, Amy L. Evans, Craig J. Sturrock, James M. Bullock, Richard D. Bardgett

**Affiliations:** 1 School of Earth and Environmental Sciences, Michael Smith Building, The University of Manchester, Oxford Road, Manchester, M13 9PT UK; 2 School of Biosciences, University of Nottingham, Sutton Bonington Campus, Loughborough, LE12 5RD UK; 3 NERC Centre for Ecology and Hydrology, Maclean Building, Benson Lane, Crowmarsh Gifford, Wallingford, Oxfordshire, OX10 8BB UK

**Keywords:** Plasticity, Roots, Drought, X-ray tomography, Grassland, Root architecture

## Abstract

**Aims:**

Root characteristics are important for predicting plant and ecosystem responses to resource scarcity. Simple, categorical traits for roots could be broadly applied to ecosystem function and restoration experiments, but they need to be evaluated for their role and behaviour under various stresses, including water limitation. We hypothesised that more complex root architectures allow more plastic responses to limited water than do tap roots.

**Methods:**

We carried out two greenhouse experiments: one with a range of grassland plant species; the other with only species of Asteraceae to test the responsiveness of root architectural classes to location of limited water in the soil column. Using trait screening techniques and X-ray tomography, we measured the plasticity of the roots in response to water location.

**Results:**

Plasticity of root biomass was lowest in tap rooted species, while fibrous and rhizomatous roots allocated biomass preferentially to where the soil was wettest. X-ray tomography indicated that root morphology was least plastic in rhizomatous species.

**Conclusions:**

Our results provide a starting point to effective categorisation of plants in terms of rooting architecture that could aid in understanding drought tolerance of grassland species. They also demonstrate the utility of X-ray tomography in root analyses.

**Electronic supplementary material:**

The online version of this article (10.1007/s11104-018-3824-1) contains supplementary material, which is available to authorized users.

## Introduction

Biomass allocation to below-ground organs could be a key process in our understanding of morphological changes in plants in response to various environmental stimuli (Freschet et al. 2015). The question of where and how plants allocate root biomass in space has contributed to uncertainty in physiological and ecosystem functioning experiments because measuring the shape, volume, and foraging behaviour of root systems is difficult and time consuming (Bardgett et al. 2014; Laliberté 2017). The challenge of characterising roots means it is very difficult to include root traits in databases, and thus to apply to real-world scenarios. One notable applied example would be ecological restoration, where practitioners are highly knowledgeable about the plant species they are using, and rely on categorical data about these species in order to successfully establish plant communities (Laughlin 2014). Inclusion of simple rooting architectural classes, underpinned by sound understanding of their potential ability to forage for limiting resources and to withstand stress could improve success rates of these endeavours. Despite these difficulties, root functional traits are increasingly being recognised as important drivers of soil functioning because they form a direct interface with soil and soil microbes, and play a key role in water and nutrient acquisition, as well as biogeochemical cycling (Bardgett et al. 2014; Bardgett 2017; Ryan and Law 2005). Root foraging behaviours and allocation patterns have mostly been tested in pot experiments, using destructive sampling followed by two-dimensional scanning to determine structural parameters (Jansen et al. 2006; Mommer et al. 2012). However, the three-dimensional nature of roots and variation in soil conditions means that in order to truly understand their role, some method of measuring morphological and physiological characteristics of roots in situ and under varying conditions is needed. To our knowledge, few studies have used 3-D X-ray scanning to characterise root morphology in an ecological context, although Paya et al. (2015) used the technique to study root competition in tree species.

In order to understand root responses to a given stress, a proxy characteristic is required that is applicable across a range of conditions, which can be used to infer the response of the species. Systematic studies are needed to test whether there is a functional basis for using categorical data, and how useful these could be, in order to address the difficulties of studying roots. Intuitively, categories based on architecture could be the most time efficient proxies, and efforts to compare their relative growth behaviours and foraging abilities could be extremely useful for a range of purposes. Freely available databases such as PLANTATT (Hill et al. 2004) assign species roots to simple categories such as tap rooted, rhizomatous, stoloniferous or fibrous. These categories are unlikely to change over environmental gradients, although their individual properties might, and thus there is an opportunity to explore the contrasting roles these different root categories may play in the soil. Tap roots are simple structures with few lateral roots and a low surface area to volume ratio. As primarily storage organs, it could be hypothesised that they contribute to soil stability (Osman et al. 2014), but are poor foragers for resources in shallow root layers (Lynch 2013) and offer fewer opportunities for microbial niches or symbioses (Schmidt and Gaudin 2017). However, they can access water in deep soil layers more effectively than shallower root systems (Alvarez-Flores et al. 2018). Stoloniferous species reproduce primarily through clonal growth, growing along the soil surface and putting out small adventitious roots, which has been shown to allow high plasticity (Song et al. 2013) and to enable resource allocation to younger clone parts when they have reached resource limited patches. By contrast, rhizomes are also clonal parts, but they lie under the soil and are more persistent than stolons, though potentially less plastic (de Kroon and Hutchings 1995). They are adapted for nutrient uptake, storing high concentrations of carbohydrates and nitrogenous reserves (Schmidt and Gaudin 2017; Suzuki and Stuefer 1999). Fibrous root systems optimise the whole soil space through vast numbers of absorptive fine roots (Campbell et al. 1991). Fibrous roots may therefore be more morphologically plastic in response to patchiness in the landscape than tap roots. Plasticity indices are already in existence and widely used (Armas et al. 2004; Navas and Garnier 2002; Valladares et al. 2006), so comparing the plasticity of different root categories is possible, but to our knowledge has not been attempted. Knowledge of the plasticity of a plant community based on the proportions of species with different root categories could offer a means of rapid assessment of its collective ability to forage for water and nutrients, and further, its potential resilience to drought stress.

We aimed to discover whether classifying plant species into simple categories of root architecture could be sufficient to understand the ability of plants to forage for limiting resources, in this case water. To address this, we carried out two experiments in pots, using similar methodologies but different plant species. The first experiment used a broad range of plant species common to temperate grassland, while the other focused on the Asteraceae family, which is an important family in these ecosystems. The second approach was used to constrain the phylogenetic variation of the species in the study. We examined the response of roots to limited water, when water was added at the top or the bottom of the pot, in contrast to when water was not limiting. The contrasting locations were designed to capture responses of the range of rooting types included in each experiment. We tested the hypothesis that more complex rooting structures with an abundance of fine roots are more able to tolerate water stress through increased plasticity, whereas species with one large tap root are likely to be less able to alter root allocation in space.

## Methods

### Experimental design

Soil was taken from an area of permanent mesotrophic grassland in Jodrell Bank, Cheshire, UK (53°13′55.6428” N, 2°18′12.8808” W). The grassland had no recent history of fertilisation or livestock grazing, and has fertile soil with vegetation dominated by *Agrostis capillaris* and *Holcus lanatus* (3.30% C; 0.23% N, pH 7.31). Soils were taken from pits up to 30 cm depth, all plant and root material was removed, and soil was homogenised and passed through a 2 mm sieve. The soil was a sandy loam, made up of 39% sand, 33% silt and 20% clay.

Both experiments used twelve plant species from temperate grasslands, which were assigned to one of four categories of root morphological traits taken from the PLANTATT database (Hill et al. 2004). These were chosen to form a gradient of rooting complexity, which would hypothetically result in contrasting responses to water location, through differences in plasticity; i.e. more complex roots mean more plasticity in response to stress. Seeds were obtained from the Millennium Seed Bank Project (RBG Kew, Ardingly, UK) and germinated on 1% agar for two weeks before being transferred to seed trays containing John Innes number 1 seed compost. When the seedlings were seven weeks old they were transferred to experimental pots to grow individually. For each experiment, there were 3 species × 4 rooting morphologies × 3 drought treatments × 4 replicates = 144 pots.

When the plants were 20 weeks old, the drought treatments commenced. For both experiments, there were three treatments: water applied to the top of the pot to 25% water holding capacity of the whole pot (WHC; hereafter TOP); water applied to the bottom of the pot to 25% WHC (hereafter BOTTOM); and a well-watered control (CONT). For the TOP and CONT treatment, water was allowed to drain freely. The CONT treatment was watered from the top of the pot, and water was continually added until saturation was observed. WHC was calculated using soil particle size and Saxton & Rawls equations (Saxton et al. 1986). We used the percentage of clay and sand to calculate the permanent wilting point (PWP; where there is no available water for plants) and field capacity (FC; where the soil is wettest without losing any to draining). The difference in soil weight between these is the water holding capacity (WHC), and so our TOP and BOTTOM treatments were created by calculating 25% of the difference between PWP and FC. We then added known weights of PWP soil to each pot and maintained each pot at the appropriate weight. Watering and weighing was carried out every two to three days to maintain water balances.

### Experiment 1: cross-taxonomy

Experiment 1 was designed to test for differences in biomass allocation among rooting morphologies across a range of plant species. The twelve plant species used were selected to cover four rooting categories, namely tap rooted, stoloniferous, rhizomatous, and fibrous. These groups were chosen because of their contrasting roles in the soil; tap roots provide stability and storage, stolons allow lateral colonisation, rhizomes optimise finding of local resource patches, and fibrous roots can fill all the local space, efficiently utilising local resources. In the tap rooted group, the species were *Daucus carota, Lotus corniculatus* and *Rumex acetosa*. For the stoloniferous group, the species were *Galium verum, Phleum pratense* and *Prunella vulgaris*. In the rhizomatous group, the species were *Plantago lanceolata*, *Poa pratensis* and *Vicia cracca.* Finally, the fibrous group comprised *Leontodon hispidus, Reseda lutea* and *Trisetum flavescens.* The pots were 20 cm deep and 8 cm across. The plants were subject to experimental drought for 55 days. At the end of the drought period the plants were immediately harvested.

At harvest, biomass was cut at the soil surface to harvest shoot biomass. The soil column was cut horizontally into four sections ~5 cm long and roots removed and treated in separate depth classes, i.e. 0–5 cm, 5–10 cm, 10–15 cm and 15–20 cm. Roots were carefully washed and biomass was weighed before and after drying at 80 °C for 24 h. The total dry weight for each plant was calculated, and the proportion of the total dry weight was calculated for each depth class. Root dry matter content (RDMC) was calculated for each depth class by dividing dry by wet weights.

### Experiment 2: asteraceae

Experiment 2 was designed to explore changes in morphology, root chemistry, and plant biomass of Asteraceae species in response to the drought treatments using a mixture of non-destructive X-ray Computed Tomography (X-ray CT) root imaging and destructive biomass measurements. The twelve Asteraceae species are common wildflowers in lowland grassland in the UK. The four rooting categories used were tap rooted, rhizomatous, ‘tap and fibrous’, and fibrous. These rooting categories conform to the main rooting morphologies found in this family: Asteraceae do not have many UK stoloniferous species, but the intermediate strategy with both tap and fibrous roots is common. In the tap rooted group the species were *Crepis capillaris, Lactuca virosa* and *Lapsana communis*. The species in the rhizomatous group were *Achillea millefolium, Leucanthemum vulgare* and *Tanacetum vulgare.* In the ‘tap and fibrous’ group, the species were *Centaurea nigra, Senecio jacobaea* and *Tragopogon pratensis*. Finally, species in the fibrous group were *Artemisia absinthium, Hypochaeris radicata,* and *Tripleurospermum inodorum*. The pots were 35 cm deep, with a diameter of 10 cm. The plants were subject to experimental drought for 55 days before being taken for 3D X-ray Computed Tomography (CT) scanning. During the scanning period the plants were maintained at either field capacity or 25% WHC as before.

### 3D x-ray computed tomography

X-ray computed tomography (X-ray CT) is a technique that allows visualisation of plant roots in situ, with the added benefit of quantification of root morphological features such as volume, root area and depth. The 3 dimensional root structure of a subset of the plants in experiment 2 was quantified using a Phoenix V|TOME|X M X-ray CT scanner at The Hounsfield Facility, University of Nottingham (GE Sensing and Inspections Technologies, GmbH, Germany, http://www.phoenix-xray.com/). To cover the full 35 cm length of the pot, three individual scans per pot were collected at 85 μm spatial resolution. Each individual scan acquired 2160 projection images (integration of two images to reduce noise) over a 360° rotation of the sample using a detector exposure time of 250 ms, at an X-ray tube voltage and current of 180 kV and 180 μA, respectively. A 0.5 mm copper filter on the X-ray tube was used to limit detector saturation issues. The resultant scan time for the three scans was 82 min. The projection images were reconstructed using Phoenix Datos version 2.0 software (GE Sensing and Inspections Technologies, GmbH, Germany). The three scan volumes were manually aligned and exported to a single volume using VGStudioMaxVersion 2.2 software (Volume Graphics GmbH, Heidelberg, Germany). The 3D structure of the roots was visualised and measured using Rootrak version 0.3.9.1 (Mairhofer et al. 2013). The measurements consisted of root volume, area, convex hull volume (smallest 3D shape the roots fit in) and depth.

The harvest was carried out as for experiment 1, although this time the roots were cut into three 10 cm sections. Additionally, in experiment 2, total carbon (C) and nitrogen (N) of dried ground root samples was also measured using an Elementar Vario EL combustion analyser (Stockport, UK), and root C and N were calculated by multiplying %C and %N by root biomass.

### Statistical analysis

Drought treatment effects on soil moisture content for each experiment were assessed using linear mixed effects models for each experiment, with root type and drought treatment as the fixed effects with an interaction term, and sampling date, pot number and species as nested random effects. All analyses were performed in R3.2.3 using the *nlme* package (Pinheiro et al. 2018).

For both experiments we carried out analyses to test whether there was a difference in root biomass across species and the drought and root architecture treatments by testing these main effects with an interaction term. We then tested whether the distribution of root biomass was altered in response to adding water in the top or the bottom of the pot by calculating the proportion of root biomass in each depth increment for each plant (5 cm increments for experiment 1 and 10 cm for experiment 2), and analysing this with species, depth, root class and drought treatment as fixed effects with all interaction terms, and pot as a random effect. The proportion of mass was arcsine square root transformed to meet assumptions of the test. Where significant effects were obtained, we used post-hoc Tukey’s HSD tests to discover which factor levels were different from one another using the lsmeans and multcomp packages in R (Lenth 2016 and Hothorn et al. 2008 respectively).

For experiment 2, we analysed the traits derived from X-ray CT (volume, root area, convex hull volume and root depth) first testing whether there was a difference in rooting type or species for these variables and if this changed with the drought treatments, with date of scanning and species as nested random effects. We tested whether these random effects were important to the model by using likelihood ratio deletion tests (LRTs), and if they were not, they were removed. The fixed effects were not simplified. We then calculated phenotypic plasticity in response to the drought treatments using Relative Distance Plasticity Indices (RDPI; Valladares et al. 2006), using the ameztegui/Plasticity function (Ameztegui 2017). The index is bounded between 0 (no plasticity) and 1 (maximum plasticity). It aims to quantify the amount of change in a given trait in response to an experimental treatment. These compared all replicates of each plant species by drought treatment for each trait, resulting in a list of values for each species. These species were then categorised by root type, and each 3D trait was analysed using lme with plant species as a random effect. All data were log transformed to meet assumptions of the tests.

## Results

### Experiment 1- cross-taxonomy

The drought treatments resulted in significant changes in soil moisture over the course of the drought period, and these were mitigated by root type (Table 1; F_6,1562_ = 12.62, *p* < 0.001). In particular, the TOP treatment resulted in lowest average soil moisture throughout both experiments. This treatment also resulted in different moisture contents for each rooting type, with moisture content being lowest in the pots containing fibrous rooted species. For CONT and BOTTOM treatments, all soil moisture was the same across root type.

**Table 1:** Average soil moisture content (%) throughout the experiment for each treatment

Rooting type	CONT	TOP	BOTTOM
Tap	10.24a	4.63b	5.01b
Rhizomatous	10.27a	4.42bc	5.67b
Stoloniferous	9.30a	3.84c	5.43b
Fibrous	9.95a	2.90d	5.12b

Letters denote significance at the *p* < 0.05 level. CONT: well-watered (control), TOP: water added in top of pots to 25% water holding capacity, BOTTOM: water added in bottom of the pots to 25% water holding capacity

**Fig. 1:**
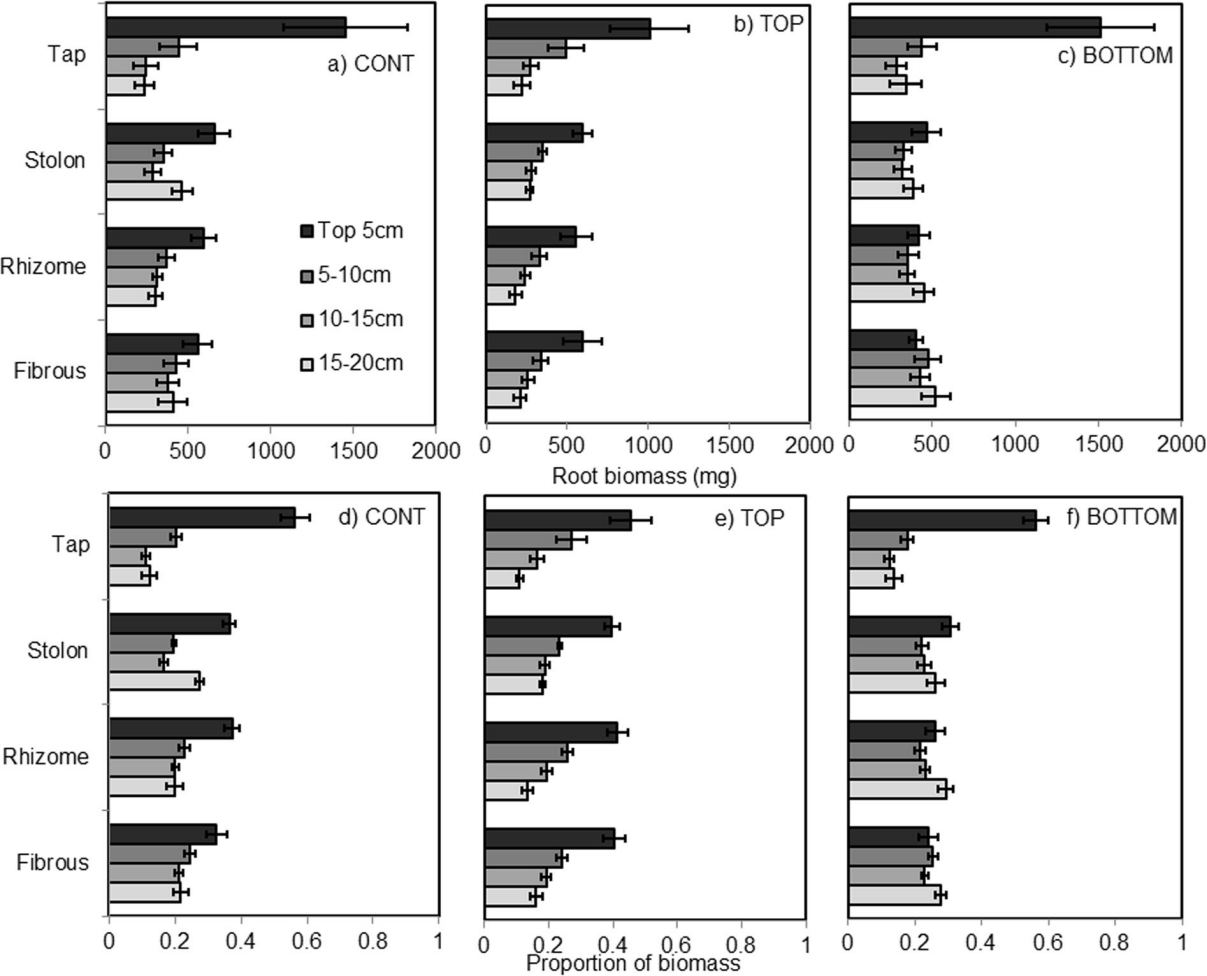
Drought and root type effects on root biomass in experiment 1, which consisted of mixed taxonomy. Vertical allocation of the total root biomass (**a**–**c**) and proportions of root biomass (**d**–**f**) for three drought treatments in 5 cm increments. CONT was well watered pots; TOP refers to water applied at the top of the pot, BOTTOM to water applied to the bottom, both of which were maintained at 25% WHC

There was no significant difference in rooting biomass across any rooting type or drought treatment, and no interaction between rooting type and drought (Fig. 1). There was, however, a significant species effect on root biomass (F_8,99_ = 23.90, *p* < 0.001; Fig. S1). Root dry matter content (RDMC) was significantly impacted by drought treatment (F_2,98_ = 20.96, p < 0.001), with the highest RDMC (densest tissues) in TOP pots (0.288 g g^−1^ ± 0.01) and the lowest in BOTTOM pots (0.212 g g^−1^ ± 0.01). RDMC was also significantly different across plant species (F_8,98_ = 10.20, p < 0.001), with the highest values occurring in the tap rooted species *Rumex acetosa* (0.325 g g^−1^ ± 0.01) and the lowest occurring in the stoloniferous species *Prunella vulgaris* (0.199 g g^−1^ ± 0.01) and rhizomatous species *Vicia cracca* (0.204 g g^−1^ ± 0.01).

Root mass distribution through the soil was significantly affected by an interaction between three variables: depth class, rooting type and drought treatment (Fig. 1; F_18,395_ = 4.91, *p* < 0.001), and also between depth class, species and drought treatment (Fig. S2; F_48,395_ = 1.80, *p* = 0.001). Under CONT conditions, most root mass was allocated mostly to the top 5 cm, with the proportion of biomass reducing steadily through the soil column. This was most pronounced in tap rooted species (Fig. 1a). Stoloniferous species showed a tendency to place a large proportion of root biomass in the bottom 5 cm of the soil column as well as the top. In the TOP treatment, this pattern was still observed, although the tap rooted species were more evenly distributed through the vertical distribution than under CONT (Fig. 1b). However, in the BOTTOM treatment, fibrous, stoloniferous and rhizomatous roots all altered their biomass so that more was allocated to deeper soil layers, resulting in approximately 25% of the biomass at every level. For fibrous and rhizomatous species, more biomass was allocated to the 15–20 cm than the 0–5 cm soil section. In contrast, tap rooted species did not alter their biomass provision to access water in the bottom of the pot (Fig. 1c).

### Experiment 2- Asteraceae

Soil moisture was highly significantly different between drought treatments (F_2,418_ = 36.73, *p* < 0.001). CONT pots were the wettest (4.63%), while TOP and BOTTOM pots were not significantly different from one another (2.24 and 1.88% respectively). Unlike in the first experiment, there was no significant difference in soil moisture content between the root types or plant species.

**Fig. 2:**
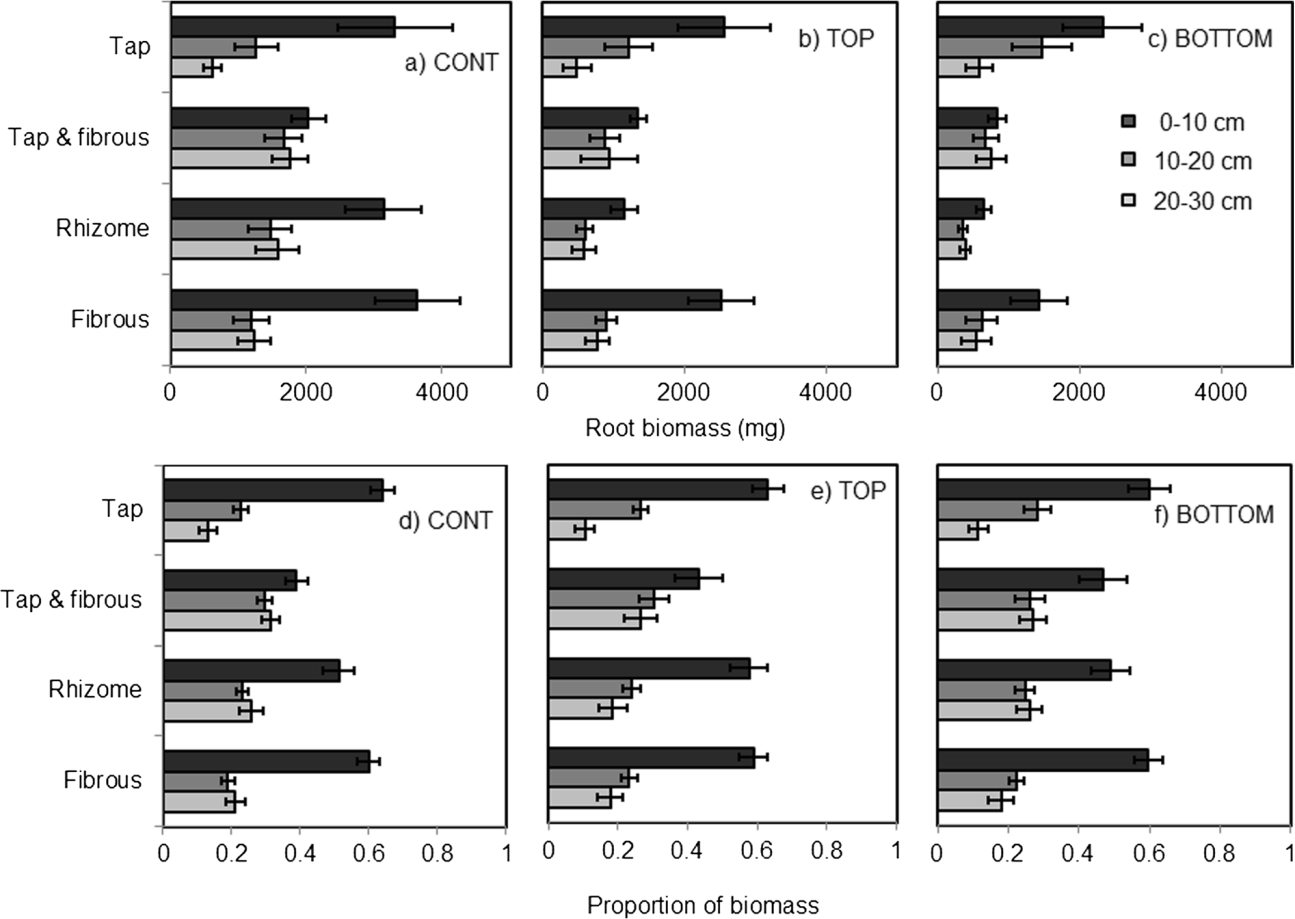
Drought and root type effects on root biomass in experiment 2, which consisted of Asteraceae species. Vertical allocation of the total root biomass (**a**–**c**) and proportions of root biomass (**d**–**f**) for three drought treatments in 10 cm increments. CONT was well watered pots; TOP refers to water applied at the top of the pot, BOTTOM to water applied to the bottom, both of which were maintained at 25% WHC

There was a significant effect of drought on root biomass of the Asteraceae species (Fig. 2 top panel; F_2,115_ = 21.88, p < 0.001; Fig. S3), whereby CONT plants had higher biomass than the other two types. This underpinned a significant interaction between drought and root type (F_6,115_ = 2.38, *p* = 0.033). Post-hoc tests revealed that for fibrous species, the BOTTOM treatment had significantly lower root biomass than the other drought treatments. For Rhizomatous species, root biomass in the CONT treatment was significantly higher than the other drought treatments. For ‘tap and fibrous’ species, CONT and BOTTOM plants were different but TOP was intermediate in biomass. Finally, there was no significant effect of drought on tap rooted mass.

In terms of vertical distribution of root biomass, there was a highly significant interaction between root type and depth class (Fig. 2a bottom panel; F_6,244_ = 10.30, *p* < 0.001), meaning that root type dictated where the plants would allocate their resources, but there was no effect of the drought treatment. The effect was driven by the main effect of depth class, which put the highest proportion of biomass in the top 10 cm of the column, while the two deeper sections (10–20 cm and 20–30 cm) were similar to each other overall. This was true of fibrous, rhizomatous and ‘tap and fibrous’ root types, which all put over 50% of their biomass into the top 10 cm of the soil column, although tap rooted species had significantly less roots allocated to each depth class in turn. There was also a significant interactive effect between plant species and drought treatment (F_22,196_ = 4.86, *p* < 0.001).

**Fig. 3:**
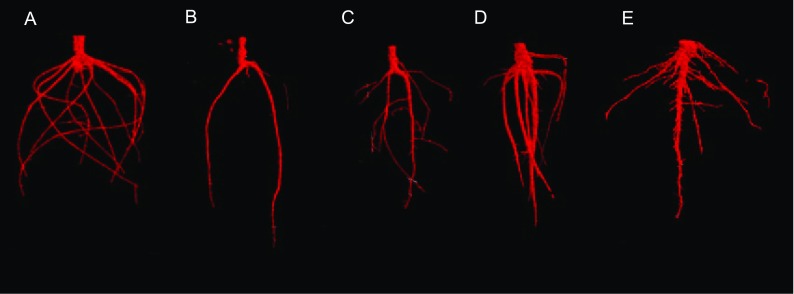
Selected images generated from 3D X-ray Computed Tomography. **a** CONT ‘tap and fibrous’ *Senecio jacobaea,* **b** BOTTOM ‘tap and fibrous’ *Tragopogon pratensis*, **c** TOP ‘tap and fibrous’ *Centaurea nigra*, **d** CONT Tap *Lactuca virosa*, **e** CONT Rhizomatous *Artemisia absinthium*

**Fig. 4:**
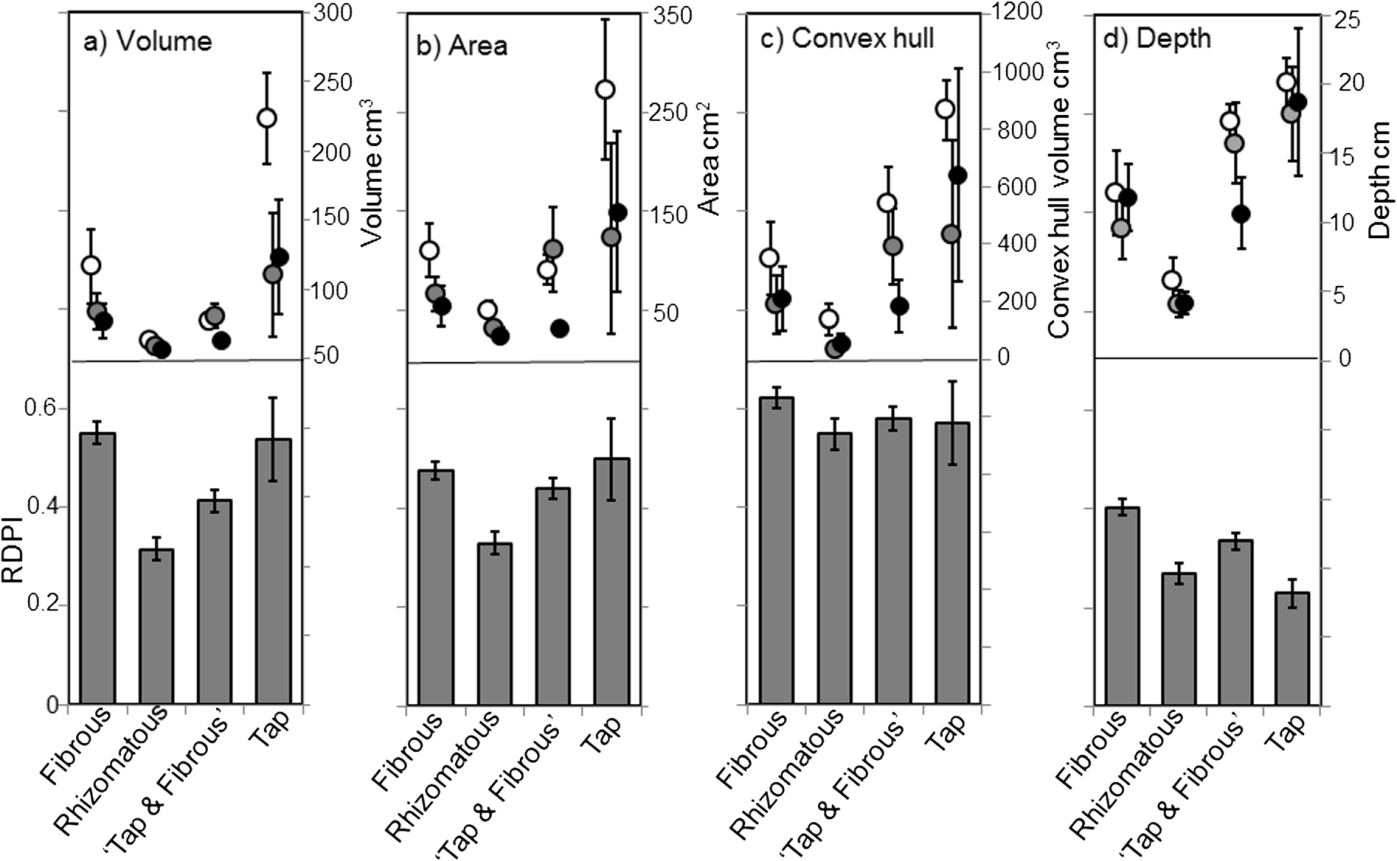
Relative Distance Plasticity Indices (RDPI) of 3D imaging of the four root structures which are corrected for species effects in the bottom panel, compared with the actual values for the three drought treatments in the top panel; CONT refers to well-watered plants (white dots), TOP refers to droughted plants where water was added to the top of the pot to 25% WHC (grey dots), which BOTTOM refers to plants where water was added to the bottom of the pots to 25% WHC (black dots)

The 3-dimensional X-ray CT scans revealed that in situ root characteristics were significantly altered by the drought treatments, and that plasticity was highly dependent on rooting type. Plasticity varied across the four variables measured (Figs. 3 and 4; Table S1). Root volume was primarily affected by the drought treatment (Fig. 4a; F_2,42_ = 13.94, *p* < 0.001), with the largest volume in the CONT pots, which was significantly different from the BOTTOM pots, and TOP was intermediate. Root type was also highly significant (F_3,42_ = 10.86, *p* < 0.001), with tap rooted species having a larger volume than any other root type. Plant species identity was also highly significant (F_6,42_ = 11.23, p < 0.001), with the largest volume being *L. virosa* and the lowest being *C. capillaris*. Date of scan comprised 7.91% of the random effect. The plasticity index (RDPI), showed that plasticity of rooting volume was highly significantly determined by root type, with the highest plasticity observed in the fibrous roots, and the lowest plasticity observed in rhizomatous roots, which were significantly different to all other types (Fig. 4a; F_3,468_ = 14.75, p < 0.001).

Root area was also primarily affected by drought treatment (Fig. 4b; F_2,43_ = 18.66, *p* < 0.001), with scan date accounting for 4.08% of the variation in the random effect, and plant species for 53.03%. Similarly to the volume, CONT and BOTTOM plants were significantly different, and TOP was intermediate. Area was significantly different across root types (Fig. 4b; F_3,43_ = 6.23, *p* = 0.001), with the largest area being in tap rooted species (17,345 cm^2^ ± 4919) and the smallest being in rhizomatous species (3529 cm^2^ ± 480). Plant species were also significantly contrasting in root area, and again, *L. virosa* and *C. capillaris* were the largest and smallest (26,531 mm^2^ and 2033 mm^2^ respectively). The RDPI was highly significantly different across rooting types (Fig. 4b; F_3,486_ = 6.07, *p* < 0.001), where tap, fibrous and ‘tap and fibrous’ roots were all significantly similar, but rhizomatous had less plastic root areas.

The convex hull was also most strongly affected by the drought treatment (Fig. 4c; F_3,43_ = 8.53, *p* < 0.001), because of a very large difference between CONT and BOTTOM. Convex hull volume was also significantly affected by root type (F_3,43_ = 4.56, *p* = 0.007), where the rhizomatous species had a different hull volume to both the tap and the ‘tap and fibrous’ species. Plant species was also significantly different (F_6,43_ = 3.30, *p* = 0.009), with *L. virosa* and *C. capillaris* having the largest and smallest convex hull volumes respectively (99,975 mm^3^ ± 140,701 and 55,095 mm^3^ ± 31,132 respectively). Date of scanning accounted for 8.22% of the variation. Plasticity of convex hull volume was not significantly different across rooting types (F_3,486_ = 1.25, *p* = 0.292).

Finally, root depth was not significantly altered by drought, but different root types had significantly different rooting depths (Fig. 4d; F_3,43_ = 13.41, *p* < 0.001). The rhizomatous species were significantly deeper than the Tap, and the ‘tap and fibrous’ species. Depth also varied significantly across plant species (F_6,43_ = 5.44, *p* < 0.001), with *L. virosa* having the deepest roots, but this time the rhizomatous species *Leucanthemum vulgare* had the shallowest (234 mm ± 19 and 35 mm ± 7 respectively), and *C. capillaris* reached an average depth close to the mean of all species, with 111 mm. Date of scanning accounted for 8.58% of the variation in the random effect. RDPI of root depth was highly significantly related to rooting type (F_3,174_ = 11.14, *p* < 0.001), with tap roots less plastic than all other types.

Total root C varied significantly with depth, with less C as roots became deeper, and this was consistent for all rooting types and drought treatments (F_2,216_ = 4.29, *p* = 0.015). There was a highly significant effect of the drought treatment on whole root system C, where C in CONT roots was higher than C in the TOP and BOTTOM types (F_2,89_ = 25.20, *p* < 0.001). RDPI of root C was significantly higher in tap rooted species than the other rooting types (F_3,1267_ = 6.95, p < 0.001), so root C was altered more in these root types in response to water limitation.

Total root N did not differ with soil depth or across root types, but there was a significant effect of the drought treatments (F_2,114_ = 6.59, *p* = 0.002), with root N being higher for the BOTTOM than the CONT treatment. However, total root N was not significantly different across root type or drought treatment. RDPI of root N was highly significantly different across root types, with the highest plasticity of root N allocation observed in Rhizomatous species, and the least in both tap and ‘tap and fibrous’ species (F_3,1267_ = 7.61, p < 0.001).

Finally root C:N ratio was not significantly different across all root types and depth classes, but was significantly lower in the BOTTOM treatment than the well-watered CONT treatment, while TOP was no different from either (F_2,114_ = 5.44, *p* = 0.006). However, there was also a significant interaction between depth class and root type, with post-hoc tests showing that this was mainly due to C:N becoming significantly higher as the roots become deeper (F_6,215_ = 2.70, *p* = 0.015). C:N ratio of the whole root system was significantly affected by the drought treatment, where BOTTOM had a lower ratio than the CONT treatment, but there was no discernible effect of rooting type (F_2,89_ = 4.25, *p* = 0.017). Plasticity of C:N showed no significant effect of the treatments.

## Discussion

We aimed to evaluate whether it was possible to use simple root categories to describe root responses to soil water limitation and location in grassland plant species. This was tested using species across a range a range of families and also within an important grassland plant family, the Asteraceae. We found that the ability of grassland plants to alter biomass allocation in response to water location was primarily dictated by rooting architectural class, but that plant species identity was also very important. This was particularly true of tap rooted species, where constituent species consistently emerged as having the largest (*L. virosa*) and smallest (*C. capillaris*) root systems, which made this group difficult to define. We also found that all species tested were able to alter their rooting depth in response to water location, but Asteraceae species were less able to alter root allocation. Those species with more complex plant rooting structures were more able to forage for water than tap rooted species. Similarly, in the Asteraceae, fibrous rooted species were the most responsive to limiting water resources in certain locations, while rhizomatous species changed their root morphology the least in response to drought, but altered their root N. We hypothesised that the likely lack of plasticity in tap roots could be due to them placing all their resources in the first growth phase, preventing plasticity in response to change. However, our results indicate that tap rooting is more likely to be a strategy that does not require plasticity in response to change and, as such, no morphological or biomass adjustments need to be made in response to water limitation.

We found that species with fibrous roots, such as *Leontodon hispidus* and *Artemisia absinthium,* had higher water uptake than those with other root forms, suggesting that foraging for water is more effective in species with finer roots. This only occurred when water was added to the top of the pot, indicating that the main foraging zone for fibrous rooted species is in the top 10 cm. However, this did not translate to higher root biomass. Fibrous roots are designed to come into contact with as much of the soil as possible, thereby maximising resource acquisition. These species are likely to be highly competitive for water, which could change community dynamics when drought occurs. Therefore, fibrous rooted species are likely to limit both root and shoot functioning in neighbouring species (Comas et al. 2013). While many fibrous rooted species are grasses, and known for being highly competitive, in our study we carefully selected a range of taxonomies for each group in order to reduce bias. Therefore, we can be confident that the observed effects are due to the rooting type and not some characteristic inherent in grass/forb/legume groups.

The cross-taxonomy study showed that when water was only available in the deepest soil layers, all root types except the tap rooted species changed their biomass allocation so that their roots were more evenly distributed through the soil column. The tap root allocations remained largely the same. This is likely to be a reflection on the adaptations of each architectural type: tap roots are designed to forage in deeper layers already so no change is necessary. In some dryland areas tap rooted species act as ‘nurse plants’, using hydraulic lift to redistribute water from deeper soil layers (Prieto et al. 2011). These species also increased their biomass when water was added to the bottom of the pots compared with other treatments, suggesting these plants direct resources towards foraging for deep water. Delgado-Baquerizo et al. (2017) found that while strong links between soil biodiversity, soil abiotic properties and plant productivity occurred in the top 10 cm of the soil, links between soil biodiversity, water availability and plant productivity were apparent up to 30 cm deep, the same depth as our mesocosms. This could be because of plasticity of plant roots as shown in our study, which may occur more readily in response to drought limitation than nutrient resource patches. More study is needed to test this idea. Some criticisms of foraging studies include the confounding factor of biomass (De Kroon and Mommer 2006). We attempted to circumvent this problem by focussing on the proportion of root biomass allocated to each section, which enables simple comparisons across species and root types.

When the Asteraceae were considered alone, they were far less able to alter biomass allocation than were those species used in the cross-taxonomy study, but their ability to make use of space in response to water location was highly plastic and dictated by root type. Biomass of tap rooted species did not change when water was limiting, indicating that fixed foraging for water in deep layers is a similar strategy across taxonomies, and that it is already optimal. Tap roots are likely to have higher hydraulic conductance due to their larger diameter which enables them to transport water to different tissues (Ho et al. 2005). Thus under drought these species are likely to be less compromised than other species. Some Asteraceae can form both tap roots and fibrous roots, so instead of stoloniferous roots that were included in the cross-taxonomy experiment, here we included a ‘tap and fibrous’ group in order to test whether this was a more successful approach in terms of growth and plasticity. This group allocated its biomass more evenly through the soil column when water was non-limiting unlike the other types. However, its plasticity was relatively low and so was its biomass, in contrast with the tap rooted species that had higher root mass under drought. Therefore, it is possible that this strategy was more optimal to access nutrients in heterogeneous environments than water. Others have suggested that this ‘dimorphism’ could be the best strategy overall, although more tests are needed to verify this idea (Ho et al. 2005). Rhizomatous species were the least plastic in terms of morphology (volume, area, depth); while the other root types were highly variable and had relatively high plasticity values. This suggests that simplicity of root architecture is not straightforwardly linked to plasticity. However, rooting architectural class does give sufficient information to be able to infer plasticity, because plasticity is closely linked with the role of the roots. Thus it should still be possible to infer the potential plasticity of a community of known rooting types based on architectural class.

Root C decreased through the soil column for all species with different root types and regardless of where the water was located. Others have postulated that location of C is linked with resource acquisition efficiency (Ho et al. 2005), and this indicates that for most species the default option is to locate resources in the top 5 to 10 cm of the soil. However, when we examined the root N in each depth increment for the Asteraceae, we found that the plasticity of N allocation was highest in the rhizomatous species, which were the least plastic in terms of biomass and morphology. Plants tend to allocate the most N to the most active roots (De Vries and Bardgett 2016), which suggests that in this group while biomass and morphology was not altered, the plants were directing resources to certain roots in response to water limitation. Changing activity is a far less risky strategy than altering biomass allocation because if conditions change and water supply changes location in the soil column, activating a different section of the roots and increasing N accordingly is likely to be less costly (Bauerle et al. 2008). This finding leads to an interesting question; should plasticity be measured in terms of activity and not just morphology? While we have not measured realised activity rates (uptake) in this study, this a potentially informative future direction. To our knowledge, there is little information on how plants alter their stoichiometry within the root in response to drought stress, although there is some information indicating that roots may become richer in non-structural carbohydrates under drought (De Vries and Bardgett 2016).

## Conclusions

In this study we have attempted to simplify the enormous array of rooting strategies in order to look for common threads. Understanding the probable outcomes of a range of scenarios based on rooting architecture, which is freely available information, is likely to be extremely valuable. Here we have made a start on this understanding using water as a limiting variable and have shown that plasticity and biomass allocation shifts in different ways according to root type, presumably to optimise limited resources. We found that tap rooted species have low ability to alter their morphology in response to small water pulses, while those root structures with a higher proportion of fine roots were able to shift the location of their roots in the soil column. This has implications for our understanding of how plant assemblages may respond to changing patterns of water availability through climate change, as well as potentially other resource types. Water limitation is a problem for many plant species, and the often unpredictable nature of localised drought means that the plant takes a risk if choosing to send roots to colonise damp soil patches (Lynch 2018). The plasticity index of the root types here allows us to begin to understand which plants will be able to withstand drought, and which are likely to gamble with their resources when accessing potentially transient water patches. Further work is needed to consider the impacts of soil types, competition and so on, but our study offers validation that this is a potentially useful area of study and could pave the way for a branch of research that uses simple categories to describe behaviour of plants under a range of stresses.

## Electronic supplementary material

ESM 1 (DOCX 420 kb)
