# Supplementary material for: Root architecture governs plasticity in response to drought
Source: Plant Soil. 2018 Oct 25;433(1):189–200. doi: 10.1007/s11104-018-3824-1 (PMC6406839; doi:10.1007/s11104-018-3824-1)
Supplement: Supplementary file 1 — (DOCX 420 kb) [file 11104_2018_3824_MOESM1_ESM.docx]

SUPPLEMENTARY MATERIAL

Figure S1:
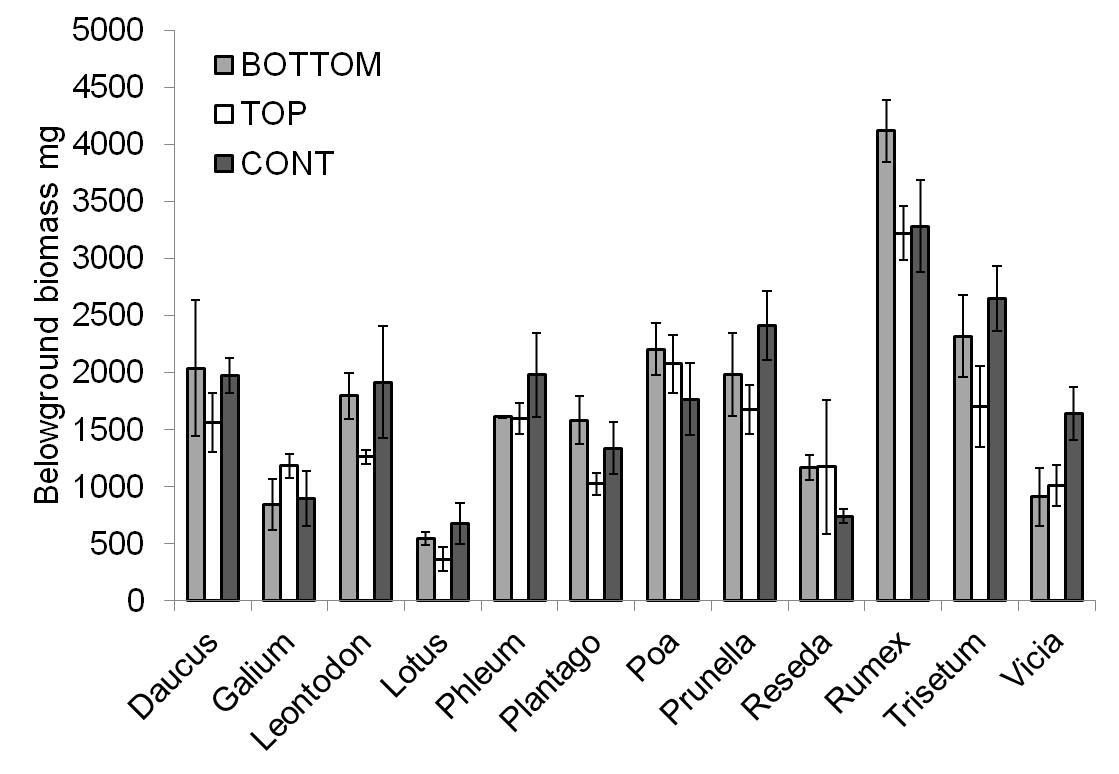
Experiment 1- species and drought effects on Belowground biomass cross-taxonomy.

Figure S2: Proportion of root mass at different column depths for the three drought treatments, divided by species.
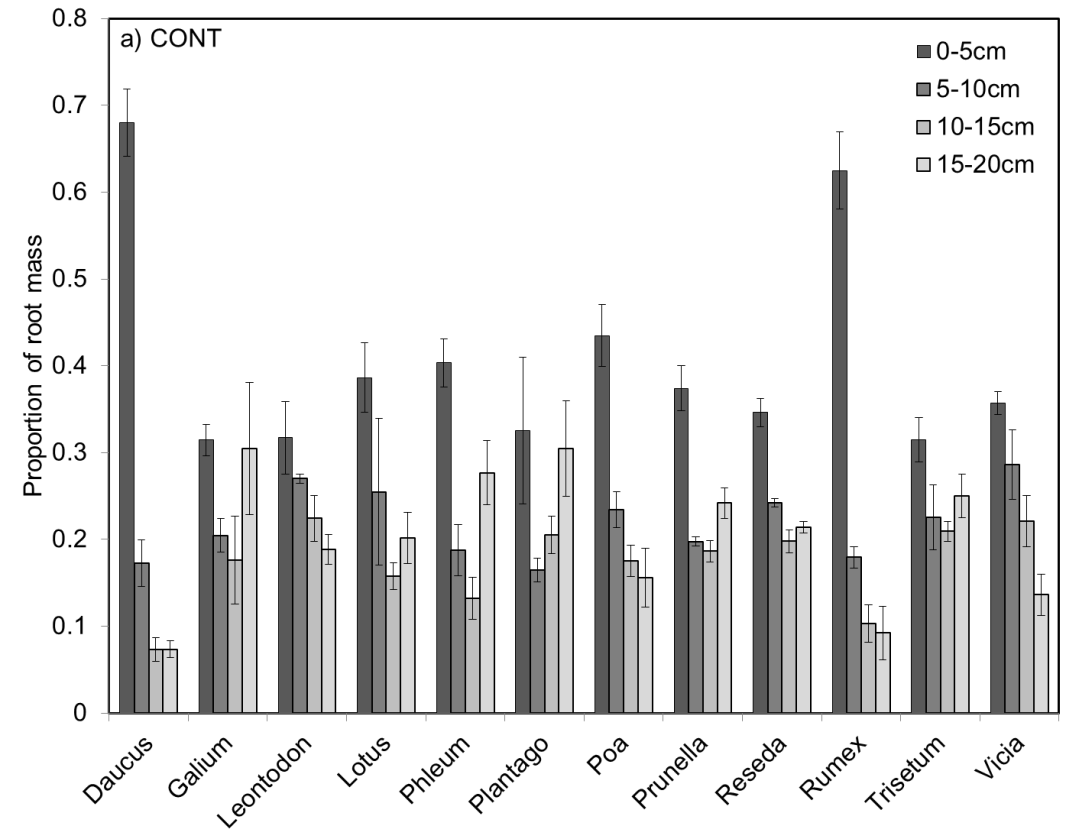


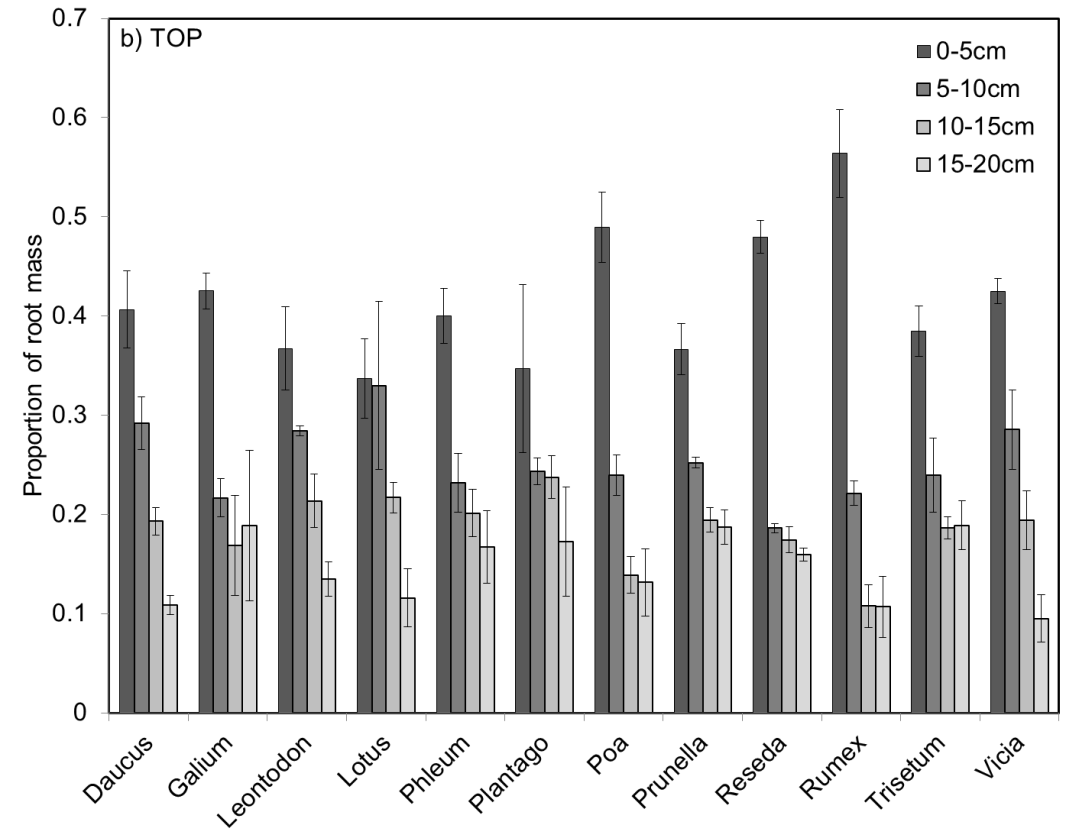


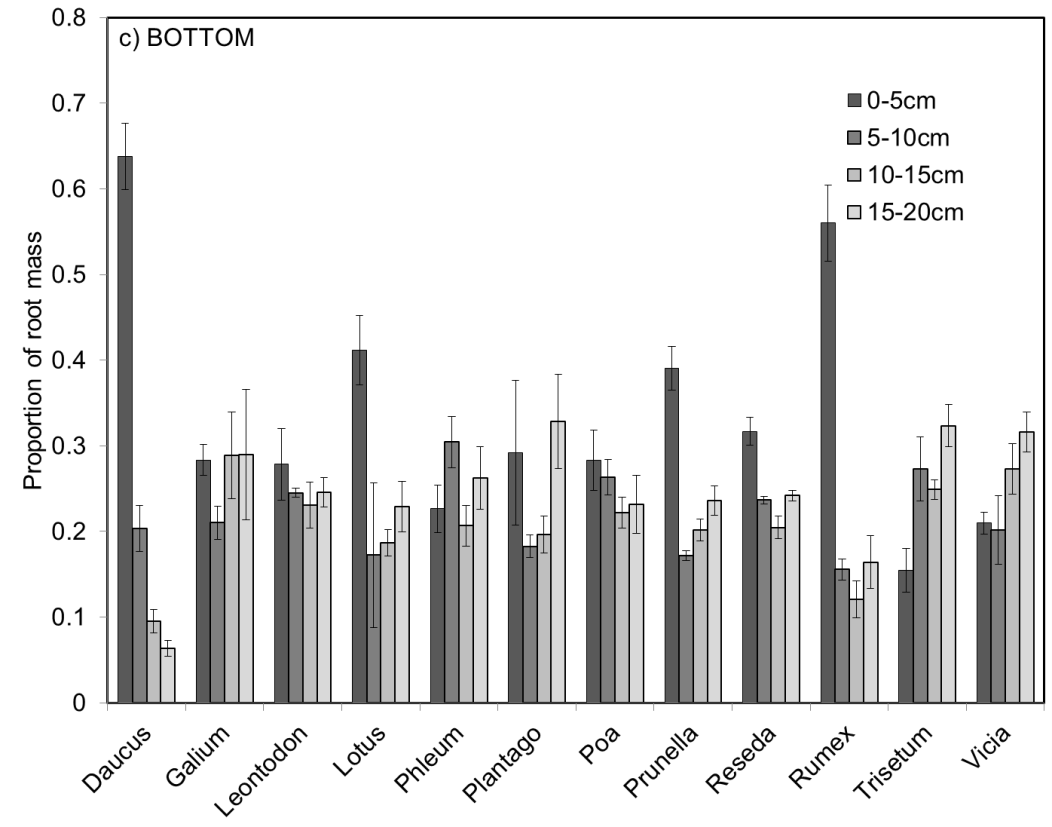


Figure S3: Experiment 2- species and drought effects on Belowground biomass of Asteraceae.


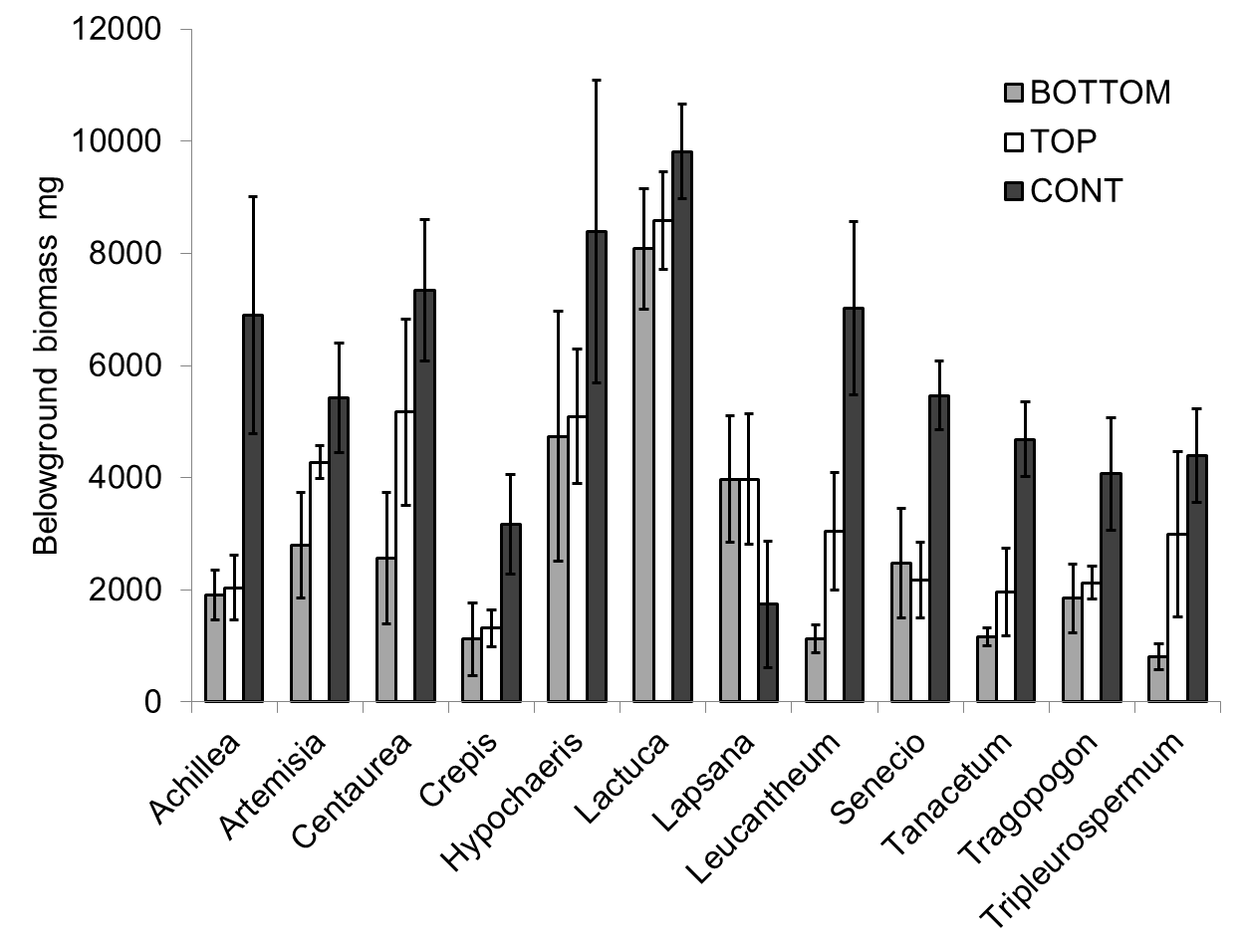


Table S1: Correlation matrix of 3D root traits in Experiment 2 (Asteraceae).

|  | Volume mm^3^ | Area mm^2^ | Convex hull volume mm^3^ | Depth mm |
| --- | --- | --- | --- | --- |
| Volume mm^3^ |  | 0.796 | 0.606 | 0.459 |
| Area mm^2^ | 0.796 |  | 0.617 | 0.514 |
| Convex hull volume mm^3^ | 0.606 | 0.617 |  | 0.594 |
| Depth mm | 0.459 | 0.514 | 0.594 |  |
